# Gypenosides ameliorate memory deficits in MPTP-lesioned mouse model of Parkinson’s disease treated with L-DOPA

**DOI:** 10.1186/s12906-017-1959-x

**Published:** 2017-09-06

**Authors:** Ting Ting Zhao, Kyung Sook Kim, Keon Sung Shin, Hyun Jin Park, Hyun Jeong Kim, Kyung Eun Lee, Myung Koo Lee

**Affiliations:** 1 Department of Pharmacy, College of Pharmacy, Chungbuk National University, Cheongju, 28644 Republic of Korea; 2 Research Center for Bioresource and Health, College of Pharmacy, Chungbuk National University, Cheongju, 28644 Republic of Korea

**Keywords:** Gypenosides, Habit learning memory, Spatial memory, MPTP-lesioned mice, L-DOPA, Parkinson’s disease

## Abstract

**Background:**

Previous studies have revealed that gypenosides (GPS) improve the symptoms of anxiety disorders in a 1-methyl-4-phenyl-1,2,3,6-tetrahydropyridine (MPTP)-lesioned rat model of Parkinson’s disease (PD). The present study aimed to investigate the effects of GPS on memory deficits in an MPTP-lesioned mouse model of PD treated with L-3,4-dihydroxyphenylalanine (L-DOPA).

**Methods:**

MPTP (30 mg/kg/day, 5 days)-lesioned mice were treated with GPS (50 mg/kg) and/or L-DOPA (10 and 25 mg/kg) for 21 days. After the final treatments, behavioral changes were assessed in all mice using passive avoidance and elevated plus-maze tests. We then evaluated the biochemical influences of GPS treatment on levels of tyrosine hydroxylase (TH), dopamine, N-methyl-D-aspartate (NMDA) receptors, extracellular signal-regulated kinase (ERK1/2), and cyclic AMP-response element binding protein (CREB) phosphorylation.

**Results:**

MPTP-lesioned mice exhibited deficits associated with habit learning and spatial memory, which were further aggravated by treatment with L-DOPA (25 mg/kg). However, treatment with GPS (50 mg/kg) ameliorated memory deficits. Treatment with GPS (50 mg/kg) also improved L-DOPA (25 mg/kg)-treated MPTP lesion-induced decreases in retention latency on the passive avoidance test, as well as levels of TH-immunopositive cells and dopamine in the substantia nigra and striatum. GPS treatment also attenuated increases in retention transfer latency on the elevated plus-maze test and in NMDA receptor expression, as well as decreases in the phosphorylation of ERK1/2 and CREB in the hippocampus. Treatment with L-DOPA (10 mg/kg) also ameliorated deficits in habit learning and spatial memory in MPTP-lesioned mice, and this effect was further enhanced by treatment with GPS (50 mg/kg).

**Conclusion:**

GPS ameliorate deficits in habit learning and spatial memory by modulating the dopaminergic neuronal and N-methyl-D-aspartate receptor-mediated signaling systems in MPTP-lesioned mice treated with L-DOPA. GPS may serve as an adjuvant therapeutic agent for memory deficits in patients with PD receiving L-DOPA.

## Background

L-3,4-Dihydroxyphenylalanine (L-DOPA), a precursor of dopamine, is the most effective therapeutic agent for controlling the motor symptoms of Parkinson’s disease (PD) [1], which mainly occur due to the loss of dopaminergic neurons in the substantia nigra pars compacta and striatum. However, chronic L-DOPA administration may lead to motor and non-motor dysfunctions, including learning and memory impairments as well as affective disorders, in both patients with PD and animal models of PD [1–3].

The dopaminergic neurons in the substantia nigra and striatum are involved in habit learning memory in humans and animals [2, 4]. L-DOPA ameliorates high-level cognitive deficits in patients with PD [4]. In contrast, L-DOPA administration has been associated with deficits in logical memory in human patients with PD [5]. In addition, N-Methyl-D-aspartate (NMDA) receptors play an important role in learning and memory, including spatial memory, in the hippocampus of humans and rats [6]. Blockage of NMDA receptors impairs learning, while excessive activation of NMDA receptors leads to the development of CNS neurotoxicity [6]. Extracellular signal-regulated kinase (ERK1/2) and cyclic AMP-response element binding protein (CREB) are also essential components of NMDA receptor-mediated signaling processes [7, 8].

*Gynostemma pentaphyllum* (Cucurbitaceae) contains approximately 90 dammarane-type triterperpene glycosides, which are collectively referred to as *Gynostemma* total saponins (gypenosides or gynosaponins; GPS) [9]. Previous studies have reported that GPS improve the symptoms of affective disorders in a 1-methyl-4-phenyl-1,2,3,6-tetrahydropyridine (MPTP)-lesioned mouse model of PD [3]. Furthermore, GPS exert protective effects against L-DOPA-induced dyskinesia in a 6-OHDA-lesioned rat model of PD [10].

Therefore, in the present study, we investigated the effects of GPS on memory deficits in an MPTP-lesioned mouse model of PD treated with L-DOPA. Following MPTP lesioning, we evaluated both behavioral changes and biochemical effects on levels of tyrosine hydroxylase (TH), dopamine, NMDA receptors, ERK1/2, and CREB phosphorylation.

## Methods

### Chemicals

GPS was purchased from Ankang Dongke Maidisen Nature Pharmaceutical Co. (purity >99%, confirmed by HPLC analysis) (Xi’an, China) [11]. L-DOPA, MPTP, dopamine, isoproterenol, and phosphor-NMDA receptor (type 1) (Ser 897) were purchased from Sigma-Aldrich (St. Louis, MO, USA). Primary antibodies against ERK1/2, and phosphor-NMDA receptor (type 1) (Ser 897) (3385S) were purchased from Sigma-Aldrich (St. Louis, MO, USA). Primary antibodies against ERK1/2 (9102S), phosphor-ERK1/2 (Thr 202/Tyr 204) (9101S), CREB (9197S), phosphor-CREB (Ser 133) (9198S), and β-actin (4967S) were purchased from Cell Signaling Tech (Beverly, MA, USA). All other chemicals were of analytical grade.

### Animals

Mice (C57BL/6, male, 20–25 g) were obtained from Samtako Co. (Animal Breeding Center, Osan, Korea) and housed at 23 ± 2 °C with 60 ± 5% humidity under a 12-h light-dark cycle with ad libitum access to water and standard diet. All procedures were approved by the Animal Ethics Committee of Chungbuk National University (Approval No., CBNUA-872-15-02), and the experiments were performed in accordance with the guidelines of the NIH for Care and Use of Laboratory Animals and Chungbuk National University Laboratory Animal Research Center.

### Experimental design

Two separate experiments were conducted using six groups (8–10 animals/group): (1) a step-through passive avoidance test including TH-immunohistochemical analysis and (2) an elevated plus-maze test including biochemical and Western blot analyses. The control group received 0.9% saline. The MPTP-lesioned group (MPTP or MPTP, 5 days) was injected with MPTP (30 mg/kg/day, daily for 5 days, i.p.) [3], which was then left for 21 days (MPTP, 26 days). The L-DOPA-treated group (MPTP + L-DOPA) was treated with L-DOPA (10 or 25 mg/kg with benserazide, 15 mg/kg, i.p., at 12:00–14:00) for 21 days following 5 days of MPTP injections. The GPS-treated group (MPTP + GPS + L-DOPA) received GPS (50 mg/kg, p.o.) for 21 days 3 h prior to L-DOPA treatment. After the final treatments, all mice were subjected to behavioral testing. The mice were then anesthetized (Zoletil 50, 100 mg/kg, i.p., Virbac, Carros, France) and sacrificed to obtain brain tissues for biochemical analyses.

### Latency time in the step-through passive avoidance test

Each mouse was placed in an illuminated chamber on the first day after the habituation period for the step-through passive avoidance test (Med Associates Inc., Vermont, USA). The initial latency time of entrance into the dark chamber was recorded, and initial latency times greater than 180 s were excluded from the experiment. Twenty-four hours later, the retention latency time was assessed as previously described [12].

### Transfer latency time in the elevated plus-maze test

The elevated-plus maze apparatus consisted of four arms (30 cm × 5 cm): two open and two closed arms of the same size, with 16-cm high black walls elevated 45 cm above the floor. The time to enter a closed arm in the first trial was recorded as the initial transfer latency. A second trial was performed 24 h after the first trial to obtain retention transfer latency. The results were expressed as the ratio of the retention transfer latency time to initial transfer latency time (%ITL) [13].

### TH immunohistochemistry

Mice were intracardially perfused with a paraformaldehyde solution (4% in 0.1 M phosphate buffered saline, pH 7.4). Coronal brain sections (30 μm) were made through the cell bodies of dopaminergic neurons of the substantia nigra (Vibratome, Leica Microsystems GmbH, Wetzlar, Germany). The sections were processed for TH-immunohistochemical staining using TH primary antibody (1:200, in 0.3% Triton X-100, Chemicon Int., Temecula, CA, USA) and biotinylated goat anti-rabbit secondary antibody (1:250; Vector Laboratories, Burlingame). Photomicrographs of TH immunoreactivity were obtained, by using the 6–10 serial sections of the substantia nigra region, following which the number of TH-immunopositive cells was determined from each mouse and the average number of the cells was counted according to a previously described method [3].

### Dopamine levels

Dopamine levels in the striatum were measured using an HPLC system (Waters 1525, Milford, MA, USA) with a Waters 120 ODS-BP column (5 μm, 150 × 4.6 mm) and an electrochemical detector (+0.85 V, Ag/AgCl reference electrode; Model 2465; Waters) as previously described [10]. Dopamine levels were expressed as a percentage of the value observed in the control group.

### Western blot analysis

Hippocampal tissues were homogenized for Western blot analysis. Phosphorylation of NMDA receptors (type 1) (Ser 897), ERK1/2 (Thr 202/Tyr 204), CREB (Ser 133), and β-actin was performed as previously described [14, 15]. Antibody binding was detected via incubation with ECL substrate (Amersham Pharmacia Biotech, Piscataway, NJ, USA) and visualized using radiographic film.

### Statistical analysis

One-way analysis of variance (ANOVA) followed by Tukey’s test was used to evaluate the effects of GPS treatment, while two-way ANOVA followed by Tukey’s test was used to analyze latency time in the passive avoidance test and to compare between the control group and MPTP-lesioned group treated with GPS and/or L-DOPA. Results were expressed as the means ± S.E.M., and *p* values <0.05 were considered statistically significant.

## Results

### Latency time in the step-through passive avoidance test

There were no differences in baseline latency times among the groups (average initial latency time, 26.6 s) (Table 1). Retention latency time was significantly shorter (decreased to 62.7 s, DF = 16, *F* = 29.5, *p* < 0.01) in the MPTP-lesioned group than in the control group (116.3 s); the time was further reduced to 57.4 s following treatment with 25 mg/kg L-DOPA (Table 1). In contrast, GPS treatment induced a significantly longer retention latency time in MPTP-lesioned mice not treated with L-DOPA (to 85.9 s; DF = 16, *F* = 19.7, *p* < 0.05) relative to the untreated MPTP-lesioned mouse group (Table 1). The MPTP-lesioned group treated with 10 mg/kg L-DOPA exhibited a significantly longer retention latency time (88.1 s; DF = 15, *F* = 21.8, *p* < 0.05) than the untreated MPTP-lesioned group, which increased further to 103.9 s (DF = 17, *F* = 17.4, *p* < 0.05) following treatment with GPS (50 mg/kg) (Table 1). The MPTP-lesioned mice treated with GPS (50 mg/kg) and L-DOPA (25 mg/kg) also exhibited a significant increase in retention latency time (increase to 76.2 s, DF = 16, *F* = 18.9, *p* < 0.05) relative to the MPTP-lesioned groups treated with L-DOPA (either dose) only (Table 1). In addition, the mean retention latency time when the MPTP-lesioned mice were grouped after any GPS and/or L-DOPA treatment was significantly shorter than that of the non-lesioned control group (DF = 52, *F* = 16.4, *p* < 0.05) (Table 1).

**Table 1:**
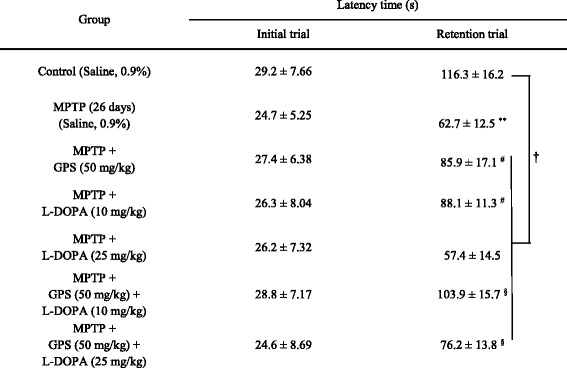
Effects of GPS on retention latency time in the passive avoidance test

After MPTP injection (30 mg/kg/day for 5 days) (MPTP), the MPTP-lesioned group was kept for 21 days (MPTP, 26 days). Treatments with GPS (50 mg/kg) and L-DOPA (10 and 25 mg/kg) in MPTP-lesioned mice and behavioral tests were performed as described in the Methods section. The results are expressed as the means ± S.E.M. for 8–10 animals per group. ^**^ *p* < 0.01 compared with the control group, ^#^ *p* < 0.05 compared with the MPTP (26 days) group, ^§^ *p* < 0.05 compared with the MPTP + L-DOPA (10 and 25 mg/kg) group, respectively (two-way ANOVA followed by Tukey’s test). ^†^ *p* < 0.05 compared between the control group and MPTP-lesioned group treated with GPS and/or L-DOPA (two-way ANOVA followed by Tukey’s test)

### Transfer latency time in the elevated plus-maze test

**Fig. 1:**
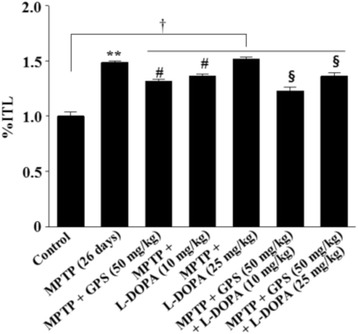
Effects of GPS on retention transfer latency time (%ITL) in the elevated plus-maze test. After MPTP injection (30 mg/kg/day for 5 days) [MPTP or MPTP (5 days)], the MPTP-lesioned group was left for 21 days [MPTP (26 days)]. Treatments with GPS (50 mg/kg) and L-DOPA (10 and 25 mg/kg) in MPTP-lesioned mice and behavioral tests were performed as described in the Methods section. The results were expressed as the ratio of the retention transfer latency time to initial transfer latency time (%ITL) and the means ± S.E.M. for 8–10 animals per group. ^**^ *p* < 0.01 compared with the control group, ^#^ *p* < 0.05 compared with the MPTP (26 days) group, ^**§**^ *p* < 0.05 compared with the MPTP + L-DOPA (10 and 25 mg/kg) group, respectively (one-way ANOVA followed by Tukey’s test). ^†^ *p* < 0.05 compared between the control group and MPTP-lesioned group treated with GPS and/or L-DOPA (two-way ANOVA followed by Tukey’s test)

The ratio of the retention transfer latency time to initial transfer latency time (%ITL) was significantly increased to 148.6% (DF = 16, *F* = 32.4, *p* < 0.01) in the MPTP-lesioned group relative to the control group; the %ITL increased further to 151.5% following treatment with L-DOPA (25 mg/kg) (Fig. 1). In contrast, MPTP-lesioned mice treated with GPS (50 mg/kg) alone significantly decreased the %ITL to 131.5% (DF = 16, *F* = 17.6, *p* < 0.05) (Fig. 1). Treatment with L-DOPA (10 mg/kg) alone reduced the %ITL to 136.2% (DF = 15, *F* = 16.3, *p* < 0.05) in the MPTP-lesioned group relative to the control group, and this value was further reduced to 123.1% (DF = 17, *F* = 17.3, *p* < 0.05) when treatment with GPS (50 mg/kg) was added (Fig. 1). Treatment with GPS (50 mg/kg) in MPTP-lesioned mice treated with L-DOPA (25 mg/kg) also reduced the %ITL to 136.1% (DF = 16, *F* = 19.1, *p* < 0.05) (Fig. 1). In addition, the %ITL (DF = 52, *F* = 11.3, *p* < 0.05) in the collected MPTP-lesioned groups (i.e. those treated with GPS and/or L-DOPA) was significantly higher than that of the control group (Fig. 1).

### TH-immunopositive cells in the substantia nigra

**Fig. 2:**
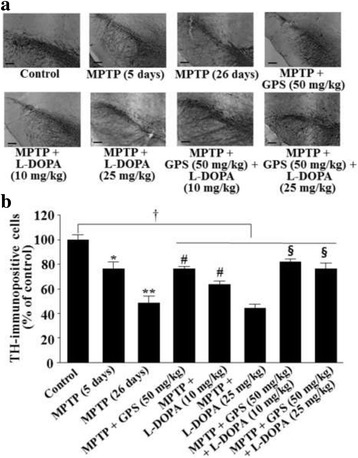
Representative photographs illustrating the effects of GPS on TH immunohistochemistry (**a**) and the number of TH-immunopositive cells (**b**) in the substantia nigra. The immunohistochemical staining analysis was performed as described in the Methods section, and images were visualized using a light microscope (100 x magnification; scale bar, 100 μm) (**a**). TH-immunopositive cells were analyzed as a percentage of the number of cells on the intact side. The number of TH-immunopositive cells in the control group was 69 ± 7 cells per section (**b**). ^*^ *p* < 0.05 compared with the control group. For further details, see Fig. 1

MPTP lesion for 5 days significantly reduced the number of TH-immunopositive cells: 76.3% of the TH cells (DF = 16, *F* = 22.6, *p* < 0.05) remained compared with the control group [3] (Fig. 2). And then, the number of TH-immunopositive cells was further reduced to 48.5% of the control number (DF = 16, *F* = 41.3, *p* < 0.01) after the 26-day-treatment with MPTP (Fig. 2). However, MPTP-lesioned mice treated with GPS (50 mg/kg) alone significantly increased the number of TH-immunopositive cells from 48.5% to 76.3% of the control group (DF = 16, *F* = 19.4, *p* < 0.05) (Fig. 2). MPTP-induced TH-immunopositive cell loss was also significantly attenuated, with 63.8% (DF = 15, *F* = 17.4, *p* < 0.05) of the control group remaining following treatment with 10 mg/kg L-DOPA; including GPS treatment (50 mg/kg), this effect was further enhanced such that the mean cell count was 81.7% of the control group (DF = 17, *F* = 18.9, *p* < 0.05) (Fig. 2). The number of TH-immunopositive cells was reduced from 48.5% to 44.3% of the control group following treatment with 25 mg/kg L-DOPA in the MPTP-lesioned group compared with the MPTP-treated group of the 26-day (Fig. 2). However, treatment with both GPS (50 mg/kg) and L-DOPA (25 mg/kg) increased the number of TH-immunopositive cells to 76.4% (DF = 16, *F* = 28.7, *p* < 0.05) of the control group (Fig. 2). In addition, the number of TH-immunopositive cells (DF = 52, *F* = 12.8, *p* < 0.05) in the collective MPTP-lesioned group treated with GPS and/or L-DOPA was significantly lower than that of the control group (Fig. 2).

### Dopamine levels in the striatum

**Fig. 3:**
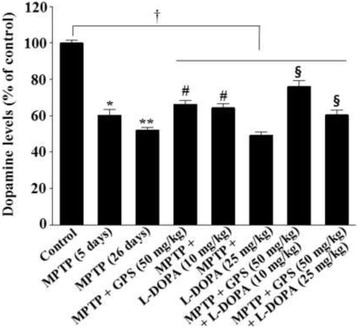
Effects of GPS on dopamine levels in the striatum. Dopamine levels were determined as described in the Methods section. Dopamine levels in the control group were 8.46 ± 0.68 ng/mg tissue. *p the slected part can be revised to < 0.05 compared with the control group. For further details, see Fig. 1

Five days of MPTP treatment significantly decreased levels of dopamine to 60.3% of the control group (DF = 16, *F* = 27.3, *p* < 0.01); dopamine levels were further decreased to 52.1% of the control group (DF = 16, *F* = 30.1, *p* < 0.01) when MPTP treatment was extended to 26 days (Fig. 3). However, dopamine levels in MPTP-lesioned mice were partially rescued by GPS treatment (50 mg/kg) to 66.3% of the control group (DF = 16, *F* = 16.0, *p* < 0.05). L-DOPA treatment (10 mg/kg) also partially rescued MPTP-induced decreases in dopamine from 52.1% to 64.4% of the control group (DF = 15, *F* = 15.9, *p* < 0.05); this value was further improved, to 76.1% (DF = 17, *F* = 13.7, *p* < 0.05), following treatment with GPS (50 mg/kg) (Fig. 3). L-DOPA treatment (25 mg/kg) reduced dopamine levels in the 26-day MPTP lesion group from 52.1% to 49.3% relative to controls (Fig. 3), whereas the combined GPS (50 mg/kg) and L-DOPA (25 mg/kg) treatment group exhibited significantly higher dopamine levels (60.5% of control levels, DF = 16, *F* = 13.4, *p* < 0.05) relative to the group treated with L-DOPA alone (Fig. 3). In addition, dopamine levels (DF = 52, *F* = 13.1, *p* < 0.05) in the collective MPTP-lesioned group treated with GPS and/or L-DOPA were significantly lower than those of the control group (Fig. 3).

### Phosphorylation of NMDA receptors, ERK1/2, and CREB in the hippocampus

**Fig. 4:**
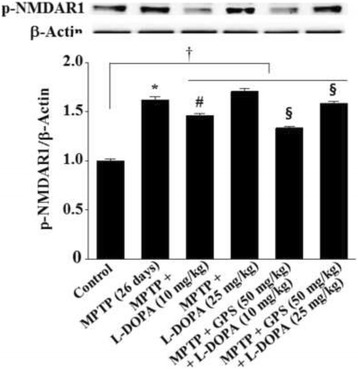
Effects of GPS on NMDA receptor (type 1) (NMDAR1) phosphorylation in the hippocampus. Phosphorylation of NMDAR1 (p-NMDAR1) and β-actin was examined as described in the Methods section. The value of the relative density ratio of the results is expressed in arbitrary units. ^*^ *p* < 0.05 compared with the control group. For further details, see Fig. 1

MPTP lesion significantly increased phosphorylation of NMDA receptors (p-NMDAR1) to 1.62-fold (DF = 16, *F* = 44.8, *p* < 0.01) more than the control group, and 25 mg/kg L-DOPA treatment further increased this to 1.71-fold more than the control group (Fig. 4). In contrast, treatment with 10 mg/kg L-DOPA significantly reduced p-NMDAR1 to 1.46-fold (DF = 15, *F* = 19.3, *p* < 0.05) more than controls, and the addition of 50 mg/kg GPS treatment further reduced this value to 1.33-fold (DF = 17, *F* = 12.7, *p* < 0.05) more than controls (Fig. 4). p-NMDAR1 in the group treated with GPS (50 mg/kg) and L-DOPA (25 mg/kg) was significantly less increased (to 1.58-fold, DF = 16, *F* = 11.5, *p* < 0.05) than that in the group treated with L-DOPA alone (Fig. 4). The p-NMDAR1 (DF = 52, *F* = 16.9, *p* < 0.05) in the collective group of MPTP-lesioned mice treated with GPS and/or L-DOPA was significantly higher than that in the control group (Fig. 4).

**Fig. 5:**
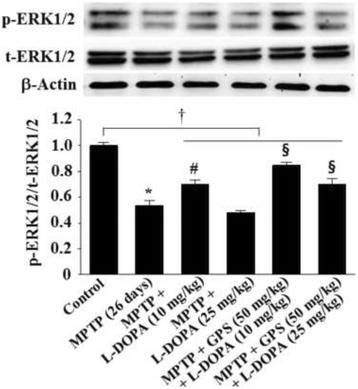
Effects of GPS on ERK1/2 phosphorylation in the hippocampus. Phosphorylation of ERK1/2 (p-ERK1/2) and total ERK1/2 (t-ERK1/2) was examined as described in the Methods section. The value of the relative density ratio of the results is expressed in arbitrary units. ^*^ *p* < 0.05 compared with the control group. For further details, see Fig. 1

In addition, MPTP administration resulted in a significant (0.53-fold) reduction in ERK phosphorylation (p-ERK1/2) (DF = 16, *F* = 30.4, *p* < 0.01) relative to controls. Treatment of MPTP-lesioned mice with 25 mg/kg L-DOPA also reduced p-ERK1/2 to 0.48-fold of controls (Fig. 5). In contrast, treatment with 10 mg/kg L-DOPA increased p-ERK1/2 to 0.71-fold (DF = 15, *F* = 18.1, *p* < 0.05) when compared with MPTP treatment alone, and this effect was further increased to 0.85-fold (DF = 17, *F* = 16.7, *p* < 0.05) of controls following treatment with GPS (50 mg/kg) (Fig. 5). MPTP-lesioned mice treated with GPS (50 mg/kg) also partially rescued p-ERK1/2 reduced by L-DOPA (25 mg/kg), with increase to 0.71-fold of controls (DF = 16, *F* = 24.3, *p* < 0.05) relative to those of the MPTP-lesioned group treated with L-DOPA alone (Fig. 5). In addition, p-ERK1/2 (DF = 52, *F* = 12.1, *p* < 0.05) in the collective group of MPTP-lesioned mice treated with GPS and/or L-DOPA was significantly lower than that of the control group (Fig. 5).

**Fig. 6:**
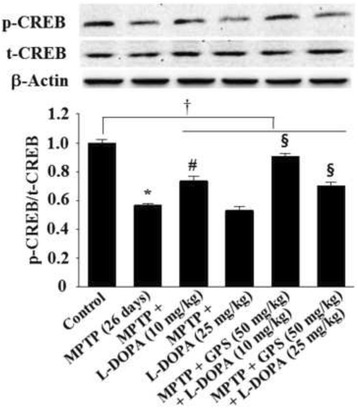
Effects of GPS on CREB phosphorylation in the hippocampus. Phosphorylation of CREB (p-CREB), total CREB (t-CREB), and β-actin was examined as described in the Methods section. The value of the relative density ratio of the results is expressed in arbitrary units. ^*^ *p* < 0.05 compared with the control group. For further details, see Fig. 1

MPTP administration also decreased CREB phosphorylation (p-CREB) to 0.56-fold (DF = 16, *F* = 27.8, *p* < 0.01) of controls, and it was further reduced to 0.53-fold following L-DOPA treatment (25 mg/kg) (Fig. 6). MPTP-lesioned mice treated with 10 mg/kg L-DOPA rescued p-CREB to 0.73-fold of controls (DF = 15, *F* = 16.6, *p* < 0.05) relative to those treated with MPTP alone; this effect was further increased to 0.90-fold (DF = 17, *F* = 17.5, *p* < 0.05) following treatment with GPS (50 mg/kg) (Fig. 6). The MPTP-lesioned mice treated with GPS (50 mg/kg) and L-DOPA (25 mg/kg) increased p-CREB to 0.71-fold of controls (DF = 16, *F* = 17.3, *p* < 0.05) relative to those treated with L-DOPA alone (Fig. 6). In addition, p-CREB (DF = 52, *F* = 10.8, *p* < 0.05) in the collective group of MPTP-lesioned mice treated with GPS and/or L-DOPA was significantly decreased relative to the control group (Fig. 6).

## Discussion

Previous studies have revealed that GPS (25 and 50 mg/kg) protect against dopamine neuronal cell death in 6-OHDA- and MPTP-lesioned animal models of PD [3, 10]. GPS (25 and 50 mg/kg) had also been observed to ameliorate symptoms of affective disorders and L-DOPA-induced dyskinesia in 6-OHDA-lesioned rats [3, 10]. In addition, GPS treatment has no significant effect on retention latency time of passive avoidance test and retention transfer latency time of elevated plus-maze test in the normal mice (control group). However, GPS (50 mg/kg) showed ameliorating effects on retention latency time of passive avoidance test and retention transfer latency time of elevated plus-maze test in MPTP-lesioned mouse model of PD (Table 1, Fig. 1). The similar results are also observed when GPS (30 mg/kg) have been treated in the MPTP-lesioned mouse model of PD [3]. In the present study, we further investigated the effects of GPS on memory deficits in MPTP-lesioned mice treated with L-DOPA, using a GPS dosage of 50 mg/kg based on the findings of these previous studies [3, 10]. The passive avoidance test is commonly used for investigating habit learning memory deficits in MPTP-lesioned mice [12], while the elevated plus-maze test is used to evaluate spatial memory [13, 16]. The biochemical influences of GPS treatment were also evaluated by comparing between the GPS + L-DOPA group and L-DOPA alone group in the MPTP-lesion group using the western blotting analysis.

MPTP injection causes dopaminergic neuronal degeneration in the substantia nigra and striatum, thereby inducing deficits in learning and memory [17]. In the present study, treatment with GPS (50 mg/kg) ameliorated decreases in retention latency time in MPTP-lesioned mice treated with L-DOPA (25 mg/kg) (Table 1). TH-immunopositive cell count and dopamine levels were decreased in the 5-day-treatment with MPTP in mice, which were further decreased in the 26-day post MPTP treatment (Figs. 2 and 3). Although the 3-day-subacute MPTP treatment may induce dopamine level recovery on the 30-day post MPTP treatment [18], subacute MPTP treatment induces dopamine neuronal lesion [19]. In addition, both TH-immunopositive cells and dopamine levels in MPTP-lesioned mice, which were further decreased following treatment with 25 mg/kg L-DOPA, were significantly improved following treatment with GPS (50 mg/kg) (Figs. 2 and 3). These results suggest that GPS ameliorate deficits in habit learning memory by preventing dopaminergic neuronal cell death in MPTP-lesioned mice.

In addition, under-excitation of NMDA receptors can produce memory dysfunctions, whereas overactivation of NMDA receptors leads to acute neuronal cell death in the CNS [6]. Previous studies have reported that the expression of NMDA receptors increases in the hippocampus following whole-brain irradiation in rats, possibly due to the death of neurons containing NMDA receptors [20]. Furthermore, NMDA receptor expression is increased in the striatal region of 6-OHDA-lesioned rats treated with L-DOPA (25 mg/kg) [21], and impairments in spatial working memory (%ITL) have been observed in both MPTP- and 6-OHDA-lesioned rats [16, 17]. These results suggest that MPTP lesioning impairs spatial memory via modulating hippocampal NMDA receptor activity, although NMDA receptor expression shows a dual pattern.

In the present study, MPTP lesioning enhanced the phosphorylation of NMDA receptors in the hippocampus, an effect that was increased by the administration of 25 mg/kg L-DOPA (Fig. 4). Conversely, MPTP lesioning reduced the phosphorylation of ERK1/2 and CREB, which was further aggravated by treatment with L-DOPA (25 mg/kg) (Figs. 5 and 6). However, treatment with GPS (50 mg/kg) partially recovered decreases in %ITL, suppressed NMDA receptor expression, and increased ERK1/2 and CREB phosphorylation in MPTP-lesioned mice treated with L-DOPA (25 mg/kg), suggesting that GPS ameliorate impairments in spatial memory by modulating the NMDA receptor-mediated signaling system.

The effects of L-DOPA on cognitive functions remain to be fully elucidated. Although low doses of L-DOPA have proven beneficial for patients with PD, long-term L-DOPA treatment may aggravate PD symptoms [1]. L-DOPA administration for 0.7–36 months may decrease TH levels in patients with PD [22]. L-DOPA administration enhances verbal learning and ameliorates high-level cognitive deficits in patients with PD [4, 23]. However, some studies have reported that L-DOPA administration results in logical memory deficits as measured using the Wechsler Memory Scale I [5]. L-DOPA administration also fails to reverse MPTP-induced memory deficits in rats [24]. In addition, long-term treatment with low doses of L-DOPA (10 mg/kg) was shown to be toxic to dopaminergic neurons of 6-OHDA-lesioned rats via the ERK-c-Jun system [14, 15]. In our experiments, MPTP-induced deficits in habit learning and spatial memory were partially recovered following treatment with L-DOPA (10 mg/kg), which has also been observed in the previous reports [25, 26], though these deficits were aggravated when higher concentrations of L-DOPA (25 mg/kg) were administered (Figs. 2 and 3). These results suggest that the neurotoxic effects of L-DOPA on cognitive functions are still complex, though they likely depend on L-DOPA concentrations. In contrast, treatment with GPS (50 mg/kg) improved both habit learning and spatial memory in MPTP-lesioned mice treated with L-DOPA (both 10 and 25 mg/kg).

L-DOPA induces oxidative cytotoxicity due to the formation of reactive oxygen species in dopaminergic neurons and PC12 cells [27]. Repeated treatments with non-toxic L-DOPA induce oxidative-induced cytotoxicity via the Epac-sustained ERK system in both dopaminergic neurons and PC12 cells [14]. Daily repeated L-DOPA administration also increases nitric oxide generation via activation of neural nitric oxide synthase [28]. Ethanol extract of *G. pentaphyllum* and GPS have protective functions against chronic stress by modulation of c-Fos expression [29]. GPS also show protective effects against oxidative damage in aortic endothelial cells [30]. In addition, previous studies have indicated that GPS protect against glutamate-induced oxidative neurotoxicity in primary cultures of rat cortical cells [11] and improve chronic cerebral hypoperfusion-induced cognitive impairments by suppressing oxidative stress in rats [31]. GPS also exert prophylactic effects on 6-OHDA-induced oxidative cell death in 6-OHDA-lesioned rats receiving long-term treatment with L-DOPA [10]. Taken together, these findings suggest that the protective functions of GPS on oxidative stress-induced cell death play a role improving habit learning and spatial memory in MPTP-lesioned mice treated with L-DOPA.

## Conclusion

Our findings indicate that GPS (25 mg/kg) exerted protective effects against both habit learning memory deficits via increasing the activation of the dopaminergic neuronal system and spatial memory deficits via modulating the phosphorylation of the NMDA receptor-mediated signaling system in an MPTP-lesioned mouse model of PD treated with L-DOPA (both 10 and 25 mg/kg). Thus, GPS may serve as an adjuvant phytonutrient for memory deficits in patients with PD receiving long-term treatment with L-DOPA.

## Abbreviations

ANOVA: analysis of variance
CNS: Central nervous system
CREB: Cyclic AMP-response element binding protein
ERK1/2: Extracellular signal-regulated kinase
GPS: Gypenosides
L-DOPA: L-3,4-Dihydroxyphenylalanine
MPTP: 1-Methyl-4-phenyl-1,2,3,6-tetrahydropyridine
NMDA: N-Methyl-D-aspartate
PD: Parkinson’s disease
TH: Tyrosine hydroxylase
